# Bilateral variant testicular arteries with double renal arteries

**DOI:** 10.1186/1757-1626-2-114

**Published:** 2009-02-02

**Authors:** Sarita Sylvia, Sridhar Varma Kakarlapudi, Venkata Ramana Vollala, Bhagath Kumar Potu, Raghu Jetti, Srinivasa Rao Bolla, Mohandas Rao, Narendra Pamidi

**Affiliations:** 1 Department of Anatomy, Mahadevappa Rampure Medical College, Gulbarga, India; 2 Melaka Manipal Medical College, Manipal, India; 3 Kasturba Medical College, Manipal, India; 4 Mamata Medical College, Khammam, India; 5 Asian Institute of Medicine, Science and Technology, Sungai Petani, Kedah, Malaysia

## Abstract

**Background:**

The testicular arteries normally arise from the abdominal aorta. There are reports about the variant origin of these arteries. Accessory renal arteries are also a common finding but their providing origin to testicular arteries is an important observation. The variations described here are unique and provide significant information to surgeons dissecting the abdominal cavity.

**Case presentation:**

During routine dissection classes of abdominal region of a 60-year-old male cadaver, we observed bilateral variant testicular arteries and double renal arteries.

**Conclusion:**

Awareness of variations of the testicular arteries such as those presented here becomes important during surgical procedures like varicocele and undescended testes.

## Background

The testicular arteries are paired vessels that usually arise from the abdominal aorta at the second lumbar vertebral level. Each artery passes obliquely downwards and posterior to the peritoneum. Descending on the posterior abdominal wall, it reaches the deep inguinal ring where it enters the spermatic cord [1,2]. There are reports about the variant origin of these arteries. Awareness of variations of the testicular arteries such as those presented here becomes important during surgical procedures like varicocele and undescended testes.

Renal arteries are a pair of lateral branches from the abdominal aorta. Normally, each kidney receives one renal artery. Variations in number, source and course of the renal arteries are common. The renal artery may give rise to branches normally derived from other vessels, such as the inferior phrenic, hepatic, suprarenal, gonadal, pancreatic and lumbar arteries [3]. Familiarity about the possible variations in the renal arterial pattern is especially important for the personnel dealing with kidney retrieval and transplantation, various endourologic procedures and innumerable interventional techniques. In most of those situations, it is the comprehensive knowledge of the renal arterial pattern which remains the key issue in determining the technical feasibility of surgical interventions as well as the post operative management [4].

## Case presentation

**Figure 1:**
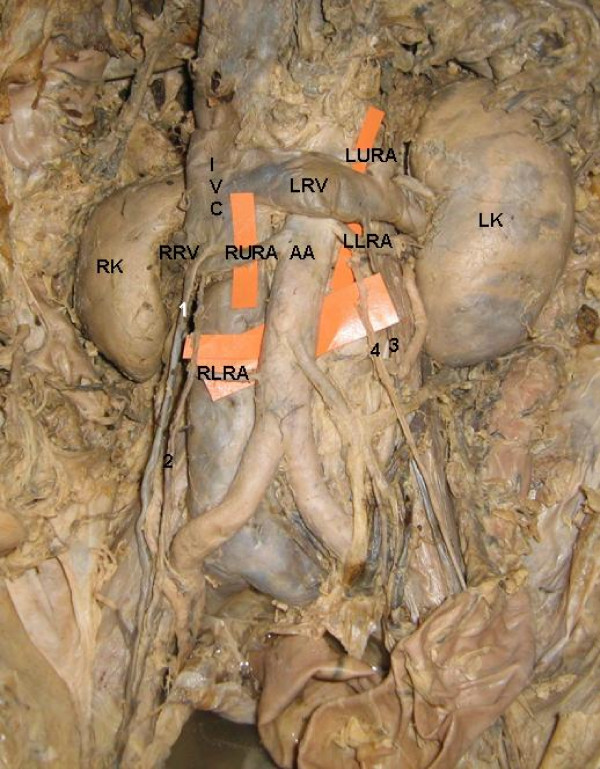
**Photograph showing variant renal and testicular arteries**. RK: right kidney, LK: left kidney, AA: abdominal aorta, RURA: right upper renal artery, RLRA: right lower renal artery, LURA: left upper renal artery, LLRA: left lower renal artery, RRV: right renal vein, LRV: left renal vein, IVC: inferior vena cava, 1: right testicular vein, 2: right testicular artery, 3: left testicular vein, 4: left testicular artery.

**Figure 2:**
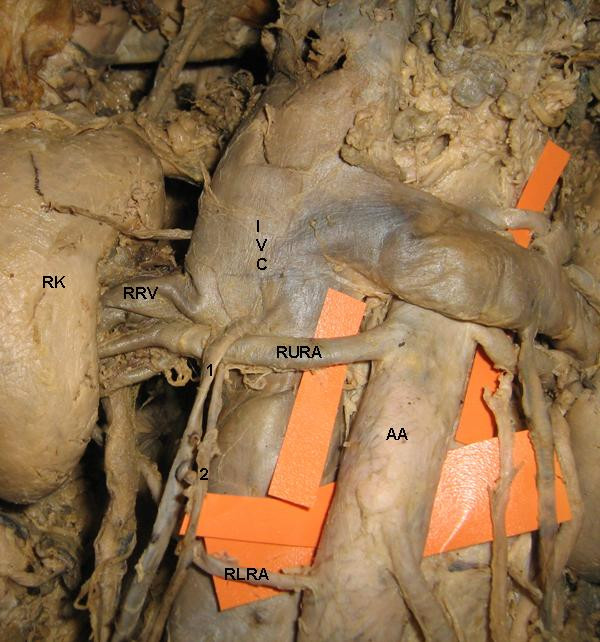
**Photograph showing double renal arteries and right testicular artery taking origin from the right upper renal artery**. RK: right kidney, AA: abdominal aorta, RURA: right upper renal artery, RLRA: right lower renal artery, RRV: right renal vein, IVC: inferior vena cava, 1: right testicular vein, 2: right testicular artery.

**Figure 3:**
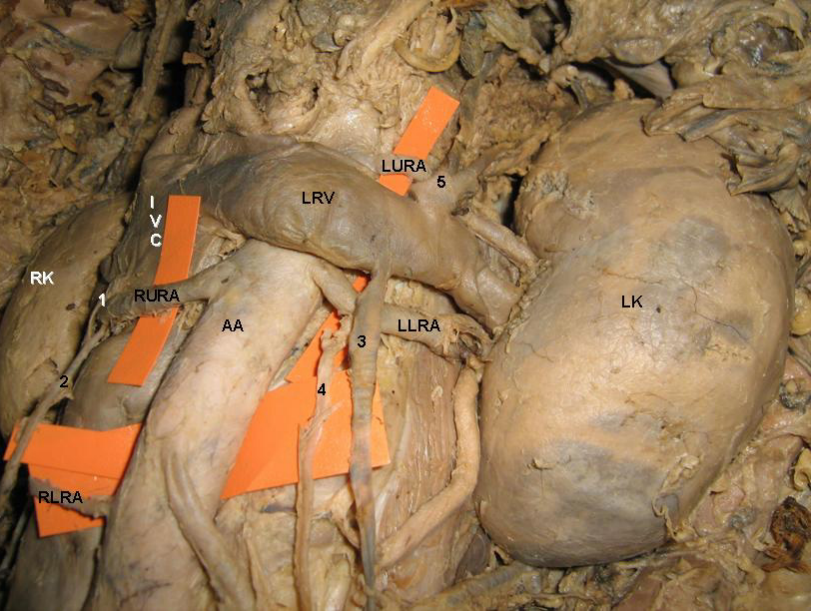
**Closure view of variant structures**. RK: right kidney, LK: left kidney, AA: abdominal aorta, RURA: right upper renal artery, RLRA: right lower renal artery, LURA: left upper renal artery, LLRA: left lower renal artery, LRV: left renal vein, IVC: inferior vena cava, 1: right testicular vein, 2: right testicular artery, 3: left testicular vein, 4: left testicular artery, 5: left suprarenal vein.

The study involved the abdominal dissection of a 60 – year – old male cadaver. The present report is about the occurrence of bilateral variant testicular arteries and double renal arteries (Figure 1). The right upper renal artery after its origin from the abdominal aorta crossed anterior to the inferior vena cava and reached the hilum of the right kidney. Near to the hilum, it provided origin to the right testicular artery (Figure 2). The right lower renal artery was coming from the lateral aspect of the abdominal aorta just above its bifurcation and crossed the inferior vena cava to reach the hilum. On the left side the left upper renal artery after its origin from the abdominal aorta passed deep to the left suprarenal vein and entered the hilum of left kidney. The left lower renal artery took origin from the abdominal aorta and passed laterally to enter the hilum of the left kidney. Before entering the hilum, it gave origin to the left testicular artery (Figure 3). The rest of the course of the testicular arteries and termination of the testicular veins was normal.

## Discussion

The anatomy of the gonadal arteries has assumed importance because of the development of new operative techniques within the abdominal cavity for operations such as varicocele and undescended testes [5]. During laparoscopic surgery of the male abdomen and pelvis many complications are due to unfamiliar anatomy in the operative field [6]. Awareness of variations of the testicular arteries, such as those shown in this case report, becomes important during such surgical procedures.

This vascular variation shows a major significance in renal surgery, in partial or total nephrectomy and in renal transplant. The presence of such variations may become a major risk when this type of gonadal artery represents the single blood supply of the gonad, without a second supply from the aorta or other arterial sources. Thus it becomes imperative to carefully preserve the gonadal artery in order to prevent any vascular troubles of the gonad, the genital artery being its unique source of blood supply. All these indicate the importance of the arteriography or Doppler ultrasound examination of the renal hilum, prior to any surgical procedure within the region [7]. A case of infarction of the left testis secondary to transcatheter embolization of a malignant left renal tumor with absolute ethanol was observed by Siniluoto et al., (1988). This is probably due to the testicular artery arising from renal artery and its branches [8].

Lippert and Pabst (1985) pointed out that the right testicular artery originated from the right renal artery in 6% of the cases [9]. In their study, Asala et al. (2001) found testicular arterial variations only on the right side in 4.7% (n = 150) of the cadavers [10]. In 4 (2.6%) of these cases, testicular arteries branched from the renal artery. Onderoglu et al. (1993) reported the right testicular artery giving rise to the inferior phrenic and the superior suprarenal arteries [11]. Cicekcibasi et al. (2002) found a gonadal artery originating from the renal artery in 5.5% of their series [12]. Although there are reports about unilateral variant origins of testicular arteries from renal arteries [13-16], bilateral variant origin of testicular arteries from additional renal arteries is rare [12].

Even though presence of accessory renal arteries is not rare globally, the testicular arteries arising from the accessory renal arteries on both sides is not common. Knowledge of this variation will help to avoid clinical complications especially during radiological examination and/or surgical approaches in abdominal region.

## Competing interests

The authors declare that they have no competing interests.

## Authors' contributions

VRV did the literature search and wrote the case report and also obtained written consent. MR and BKP conceived the study and helped to draft the manuscript. SRB, SvK, RJ, SS and NP helped in the literature search. All authors had gone through the final manuscript and approved it.

## Consent

Written informed consent was obtained from the subject's relative for publication of this case report.

## References

[B1] Hollinshead WH Anatomy for Surgeons 1971 2 New York: Harper and Row 579 5805163375

[B2] Moore KL Dalley AF Clinically oriented anatomy 1999 4 Philadelphia: Lippincott Williams and Wilkins 29211541844

[B3] Bergman RA Thompson SA Afifi AK Compendium of Human Anatomic Variations 1988 Munich: Urban and Schwarzenberg 81

[B4] Pushpa Dhar Kumud Lal Main and accessory renal arteries – A morphological study It j anat embryol 2005 110 101 110 16277160

[B5] Brohi RA Sargon MF Yener N High origin and unusual suprarenal branch of a testicular artery Surg Radiol Anat 2001 23 207 208 10.1007/s00276-001-0207-7 11490935

[B6] Cussnot O Desgrandehamps F Bassi S Teillae P Lassau JP Le Due A Anatomic basis of laproscopic surgery in the male pelvis Surg Radiol Anat 1993 15 265 269 10.1007/BF01627877 8128333

[B7] Bordei Petru Sapte Elena Iliescu Dan Dina Constantin The morphology and the surgical importance of the gonadal arteries originating from the renal artery Surg Radiol Anat 2007 29 367 371 10.1007/s00276-007-0224-2 17593308

[B8] Siniluoto TM Hellstrom PA Paivansalo MJ Leinonen AS Testicular infarction following ethanol embolization of a renal neoplasm Cardiovasc Intervent Radiol 1988 11 3 162 164 10.1007/BF02577110 3139298

[B9] Lippert H Pabst R Bergman JF Arterial Variations in Man, Classification and Frequency 1985 Verlag, Munchen 25 29

[B10] Asala S Chaudhary SC Masumbuko-Kahamba N Bidmos M Anatomical variations in the human testicular blood vessels Ann Anat 2001 183 545 549 11766526 10.1016/S0940-9602(01)80064-9

[B11] Onderoglu S Yuksel M Arik Z Unusual branching and course of the testicular artery Anat Anz 1993 175 541 544 10.1016/s0940-9602(11)80219-0 8297041

[B12] Cicekcibasi AE Salbacak A Seker M Ziylan T Buyukmumcu M Uysal II The origin of gonadal arteries in human fetuses: anatomical variations Ann Anat 2002 184 3 275 279 12056759 10.1016/S0940-9602(02)80126-1

[B13] Deepthinath R Satheesha Nayak B Mehta RB Bhat S Rodrigues V Samuel VP Venkataramana V Prasad AM Multiple variations in the paired arteries of the abdominal aorta Clin Anat 2006 19 6 566 568 10.1002/ca.20207 16283657

[B14] Okamoto K Kodama K Kawai K Wakebe T Saiki K Nagashima S The inferior supernumerary renal arteries: a classification into three types Ann Anat 2006 188 1 49 53 16447912 10.1016/j.aanat.2005.06.004

[B15] Bergman RA Cassell MD Sahinoglu K Heidger PM Jr Human doubled renal and testicular arteries Ann Anat 1992 174 4 313 315 1416060 10.1016/s0940-9602(11)80292-x

[B16] Notkovich H Variations of the testicular and ovarian arteries in relation to the renal pedicle Surg Gynecol Obstet 1956 103 4 487 495 13360658

